# Pathological modelling of pigmentation disorders associated with Hutchinson-Gilford Progeria Syndrome (HGPS) revealed an impaired melanogenesis pathway in iPS-derived melanocytes

**DOI:** 10.1038/s41598-018-27165-y

**Published:** 2018-06-14

**Authors:** Alessandra Lo Cicero, Manoubia Saidani, Jennifer Allouche, Anne Laure Egesipe, Lucile Hoch, Celine Bruge, Sabine Sigaudy, Annachiara De Sandre-Giovannoli, Nicolas Levy, Christine Baldeschi, Xavier Nissan

**Affiliations:** 1 CECS, I-Stem, Corbeil-Essonnes, 91100 France; 2 INSERM U861, I-Stem, Corbeil-Essonnes, 91100 France; 3 UEVE U861, I-Stem, Corbeil-Essonnes, 91100 France; 4 Aix Marseille Univ, INSERM, MMG, Marseille, France; 5 Department of Medical Genetics, Children’s Hospital La Timone, Marseille, France

## Abstract

Hutchinson-Gilford Progeria Syndrome (HGPS) is a rare genetic disorder that leads to premature aging. In this study, we used induced pluripotent stem cells to investigate the hypopigmentation phenotypes observed in patients with progeria. Accordingly, two iPS cell lines were derived from cells from HGPS patients and differentiated into melanocytes. Measurements of melanin content revealed a lower synthesis of melanin in HGPS melanocytes as compared to non-pathologic cells. Analysis of the melanosome maturation process by electron microscopy revealed a lower percentage of mature, fully pigmented melanosomes. Finally, a functional rescue experiment revealed the direct role of progerin in the regulation of melanogenesis. Overall, these results report a new dysregulated pathway in HGPS and open up novel perspectives in the study of pigmentation phenotypes that are associated with normal and pathological aging.

## Introduction

Hutchinson-Gilford Progeria Syndrome (HGPS) is a rare genetic disorder that leads to premature aging. In its classical form, this syndrome is caused by a de novo single-base substitution c.1824 C > T in the LMNA gene^1,2^. Even though the mutation is silent (G608G), it activates an alternative cryptic splicing site and the production of a truncated and constitutively farnesylated form of lamin A, called progerin^3^. In patients with progeria, the accumulation of this toxic protein triggers a spectrum of symptoms that resemble aging, with lipodystrophy, dermal and bone abnormalities and cardiovascular alterations, leading to a shortened lifespan^4^. Over the past 10 years, several therapeutic strategies have been proposed to treat such patients, either by inhibiting progerin farnesylation with farnesyl transferase inhibitors^5–7^, statins and bisphosphonates^8^, or by decreasing its production using morpholinos^9^, rapamycin^10^, retinoids^11–13^ or, more recently, with metformin^14^ and MG132^15^.

Nuclear lamins are the major structural proteins of the nuclear lamina^16,17^. Since the discovery of the molecular mechanism underlying this syndrome, several studies have highlighted the major role of progerin in the dysregulation of key biological processes, such as cell proliferation, cell differentiation, the DNA repair process or mitochondrial activities^3^. The development of relevant cellular models for HGPS to study these biological dysfunctions has been a major challenge over the past decade. In this context, it is noteworthy that the breakthrough discovery of induced pluripotent stem cells (iPS cells) by Shinya Yamanaka has been a unique tool in the discovery of new dysregulated pathways and the identification of biological targets^18^. Thanks to their pluripotency and self-renewal properties, iPS cells allowed access to cell types that were relevant, but still unexplored in specific diseases^19^. In 2011, several research groups demonstrated the capacity of iPS cell lines to recapitulate some aspects of HGPS after differentiation into vascular smooth muscle cells (VSMCs)^20,21^ and mesenchymal stem cells (MSC). In 2012, our group used the same model to report the absence of progerin expression in neurons and the role of a neuron-specific microRNA, miR-9 in the preservation of cognitive capacities in patients^22^. More recently, two studies have revealed the effect of progerin on adipogenic^23^ and osteogenic differentiation^13^ processes of MSCs derived from HGPS iPS cells.

In this study, we used iPS cells derived from HGPS patients to study pigmentation disorders associated with this premature aging syndrome. To do so, we took advantage of the recent development of an efficient protocol of differentiation, allowing the generation of a pure population of functional melanocytes from pluripotent stem cells. The first method of differentiation was described in 2006, reporting an efficient method for differentiating hESCs into a melanocyte population within 4–6 weeks through the generation of embryoid bodies and their treatment using three growth factors: Wnt3a, endothelin-3, and stem cell factor^24^. In 2011, our group reported an alternative 2D protocol of differentiation, allowing the generation of melanocytes from both hESC and iPSC^25^. In addition, we reported that these cells could be used to study pathological conditions, as demonstrated for neurofibromatosis type 1^26^. In the present study, we have used this model to investigate the role of progerin in the depigmentation observed in HGPS patients.

## Results

**Figure 1:**
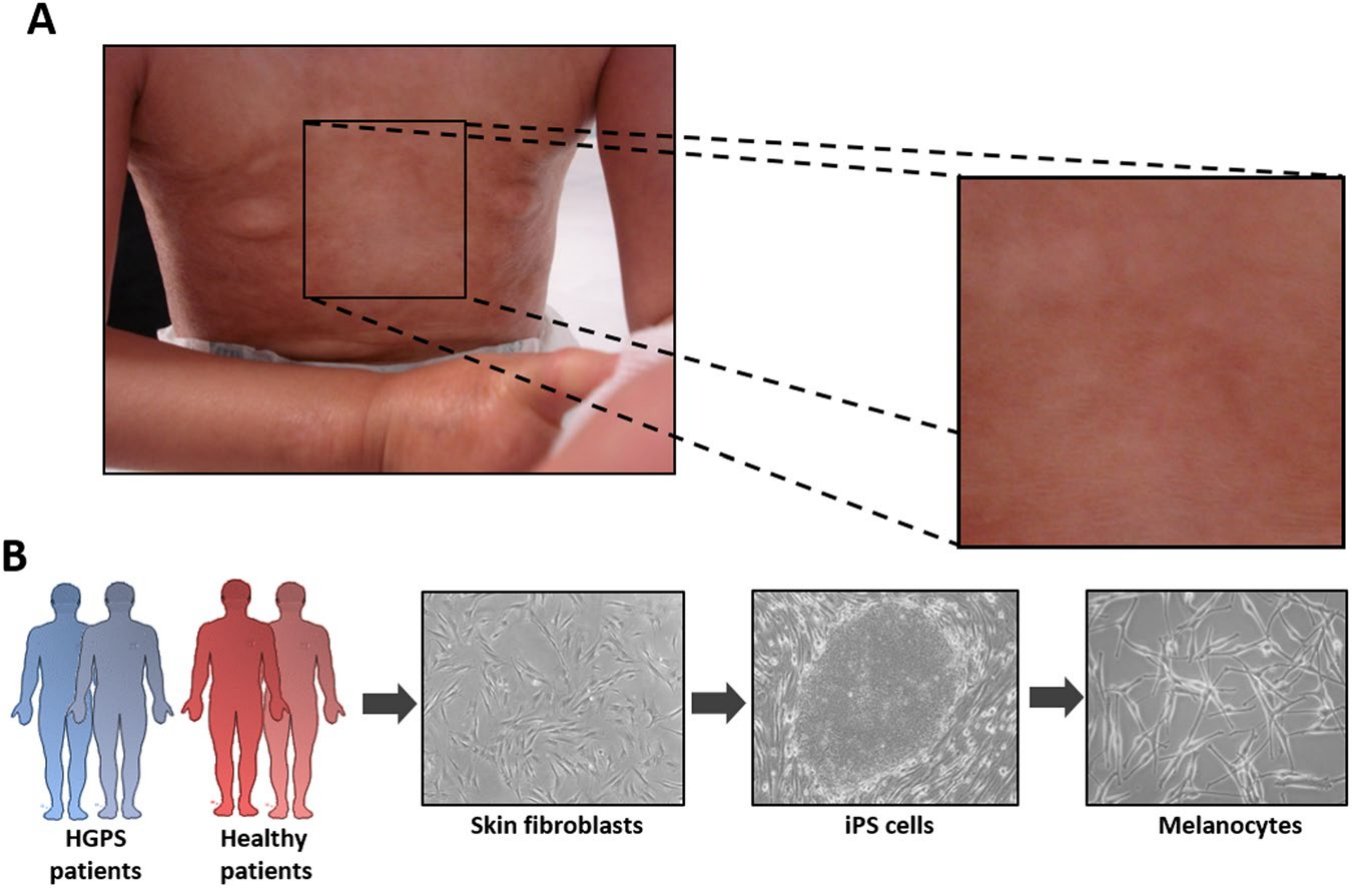
Pathological modelling of pigmentation disorders in HGPS patients. (**A**) Picture of the skin of a HGPS patient that shows global hypopigmented skin, with a typical mottled pigmentation that alternates between hypo- and hyper-pigmented skin areas. The patient also shows typically prominent nipples, due to altered subcutaneous adipose tissue distribution. (**B**) Schematic representation of the pathological modelling strategy to model pigmentation disorders in HGPS patients.

**Figure 2:**
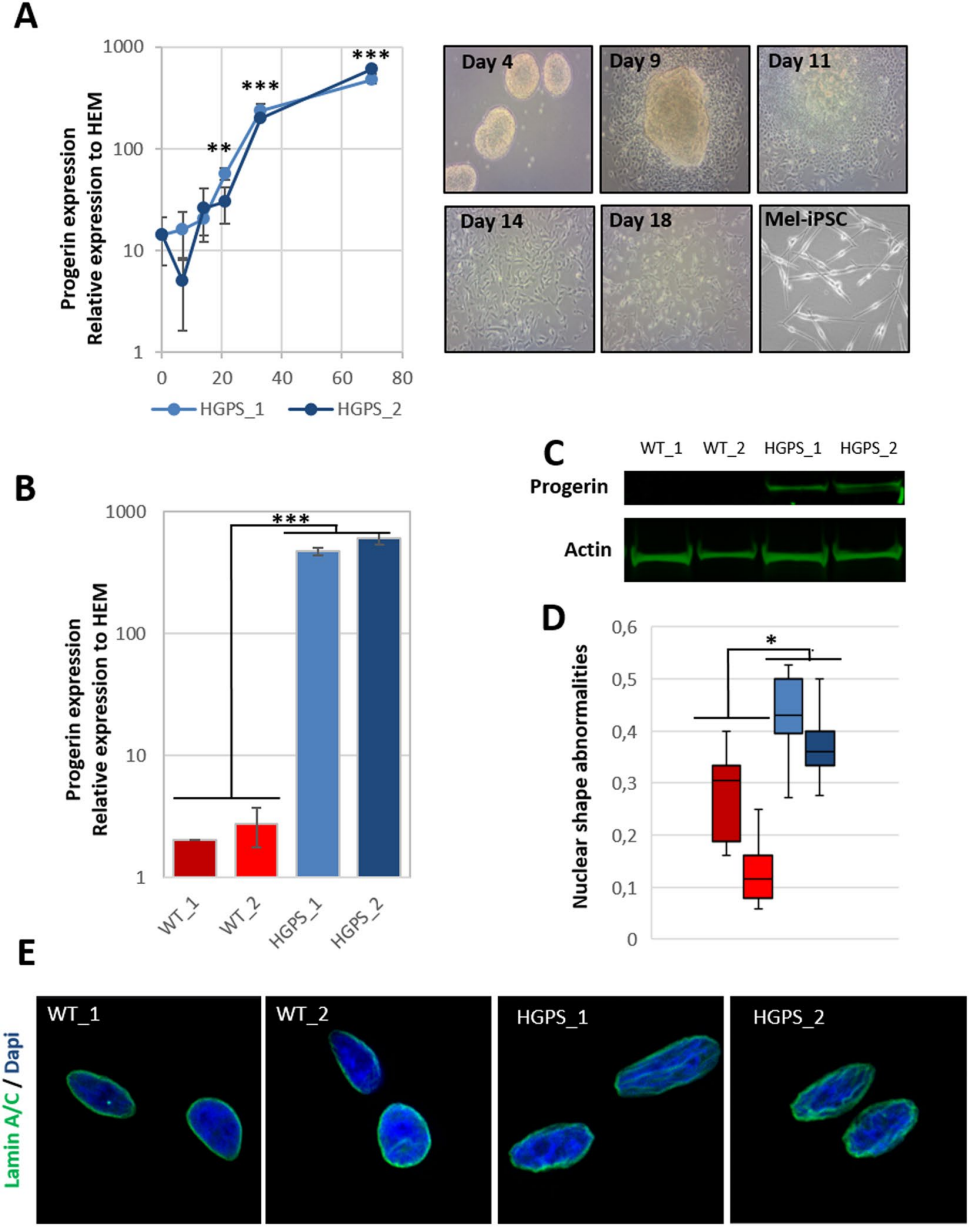
Measurement of progerin expression and nuclear shape abnormalities in WT and HGPS melanocytes. (**A**) Measurement of progerin expression in HGPS iPS during the melanogenic differentiation process at 0, 7, 14, 21, 28 and 70 days (equivalent to melanocyte passage 4). Human epidermal melanocytes (HEM) were used as a control. Statistical analysis was performed with one-way analysis of variance (ANOVA), using Dunnet’s comparison test. Values of p < 0.05 were considered significant (*p < 0.05, **p < 0.01, ***p < 0.001). Representative pictures of the process of differentiation of iPS cells into Mel-iPSC at 4, 9, 11, 14, 18 days. (**B**) Comparative gene expression analysis for progerin in two WT (WT_1 and WT_2) and two HGPS (HGPS_A and HGPS_2) Mel-iPSCs. HEMs were used as a control. Statistical analysis was performed with one-way analysis of variance (ANOVA), using Dunnet’s comparison test. Values of p < 0.05 were considered significant (*p < 0.05, **p < 0.01, ***p < 0.001). (**C**) Western blot analysis of progerin expression in WT and HGPS Mel-iPSCs. Actin was used as a control. The grouping of gels/blots was done from different gels. Uncropped gels are presented in sup figure (**A**,**B**). (**C**) Quantification of nuclear shape abnormalities in WT and HGPS Mel-iPSCs. Statistical analysis was performed with one-way analysis of variance (ANOVA), using Dunnet’s comparison test. Values of p < 0.05 were considered significant (*p < 0.05, **p < 0.01, ***p < 0.001). (**D**) Lamin A/C immunostaining in WT and HGPS Mel iPSCs, showing typical nuclear disorganization of nuclear shape abnormalities.

**Figure 3:**
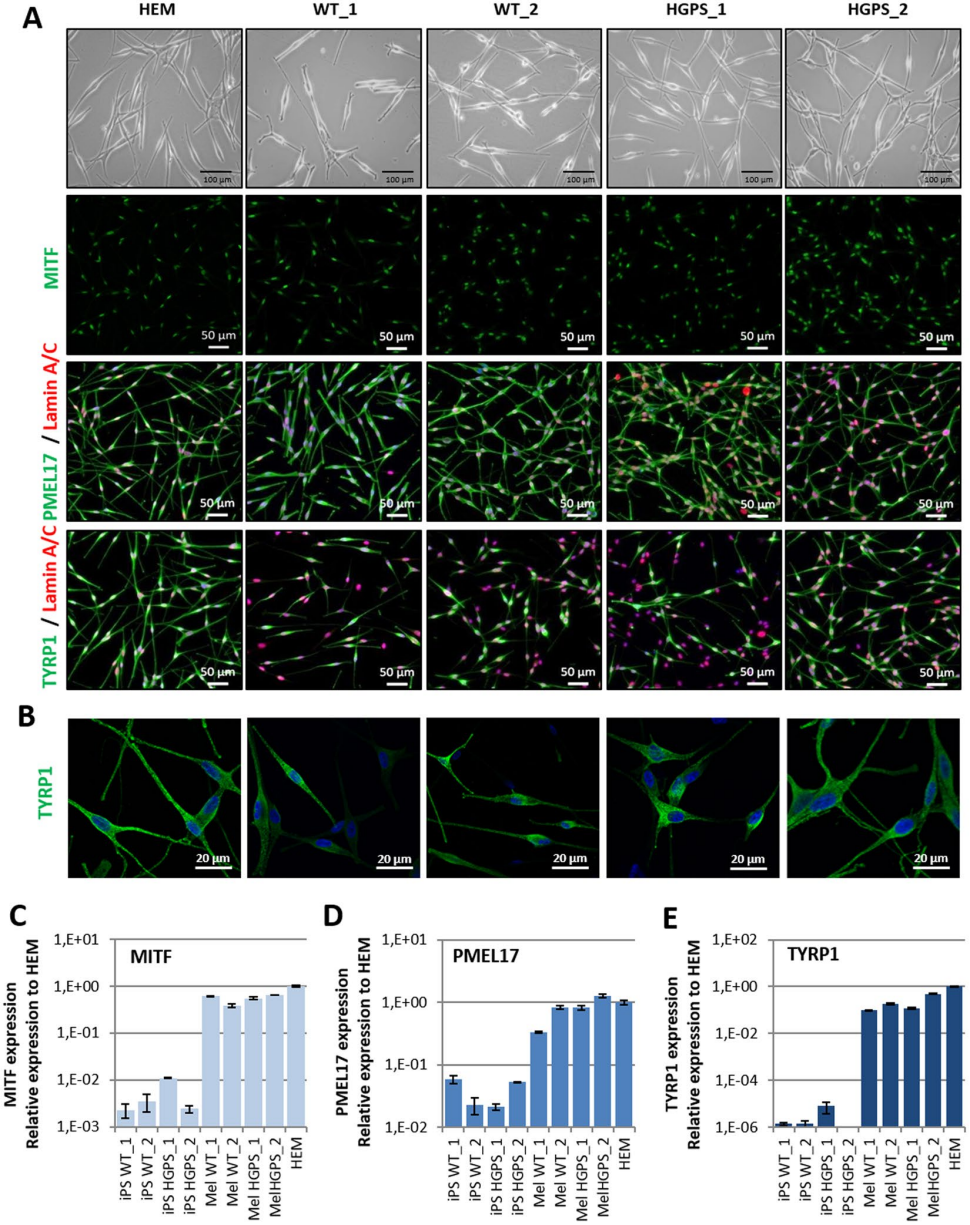
Molecular characterization of melanocytes derived from iPS cells. (**A**) MITF, PMEL17, TYRP1 (green) and Lamin A/C (red) immunostaining in WT (WT_1 and WT_2), HGPS (HGPS_1 and HGPS_2) Mel-iPSC and HEM cells. (**B**) Confocal analysis of TYRP1 localization in WT (WT_1 and WT_2), HGPS (HGPS_1 and HGPS_2) Mel-iPSC and HEM cells. (**C**) Gene expression analysis of MITF in WT (WT_1 and WT_2), HGPS (HGPS_1 and HGPS_2) iPS, Mel-iPSC and HEM cells. HEMs were used as a control. (**D**) Gene expression analysis of PMEL17 in WT (WT_1 and WT_2), HGPS (HGPS_1 and HGPS_2) iPS, Mel-iPSC and HEM cells. HEMs were used as a control. (**E**) Gene expression analysis of TYRP1 in WT (WT_1 and WT_2), HGPS (HGPS_1 and HGPS_2) iPS, Mel-iPSC and HEM cells. HEMs were used as a control.

Dermatological observations on the skin of HGPS patients reveal global hypopigmentation, associated with a typical mottled pigmentation (Fig. 1A). To investigate the molecular mechanism underlying these symptoms, two iPS cell lines derived from two HGPS patients (HGPS1 and HGPS2) and two healthy patients (WT1 and WT2) were differentiated into melanocytes using a protocol of differentiation based on the ones described by Allouche *et al*.^26^, Gledhill *et al*.^27^ and Mica *et al*.^28^ (Fig. 1B). Briefly, embryoid bodies (EBs) were generated in low attachment conditions and committed for 4 days in the neural crest lineage. Once generated, EBs were seeded on dishes coated with 0.1% gelatin for 3 days and in a melanogenic differentiation medium for 11 days. At this stage, differentiating cells appeared with a morphology typical of a melanocytic lineage migrated out of adherent EBs by day 9. At day 18, iPS-derived melanocytes (Mel-iPSC) present a melanocyte-like morphology and can be cultivated in melanocyte culture medium for several passages. No difference was observed in HGPS and WT cells in terms of neural crest commitment or differentiation rate. Progerin expression was monitored by qPCR during the process of melanogenic differentiation, revealing no significant difference between WT and HGPS cell lines before 8 days of differentiation (Fig. 2A). Kinetics of differentiation revealed an increase in expression of progerin over time and stabilization after four passages of Mel-iPSC (Day 35) in culture (Fig. 2B). All further experiments were carried out in this passage. Progerin expression in Mel-HGPS was confirmed by western blot at the protein level (Fig. 2C, uncropped blots in sup Fig A and B). Nuclear shape disorganization of Mel-HGPS was measured by quantifying the percentage of abnormal lamin A/C-positive nuclei, revealing a significant increase in abnormal nuclei in the two HGPS cell lines (Fig. 2D and E). Observations of the cells obtained using this method revealed no difference between HGPS, WT and adult melanocytes in terms of morphology (Fig. 3A). Immunostaining of lamin A/C confirmed that all the differentiated cells express lamin A/C (Fig. 3A). Finally, molecular characterization of the expression of the key melanogenic markers, MITF, PMEL17 and TYRP1, in these cells revealed no differences in terms of expression of these proteins (Figs 2B and 3A) and mRNA (Fig. 3C,D and E).

**Figure 4:**
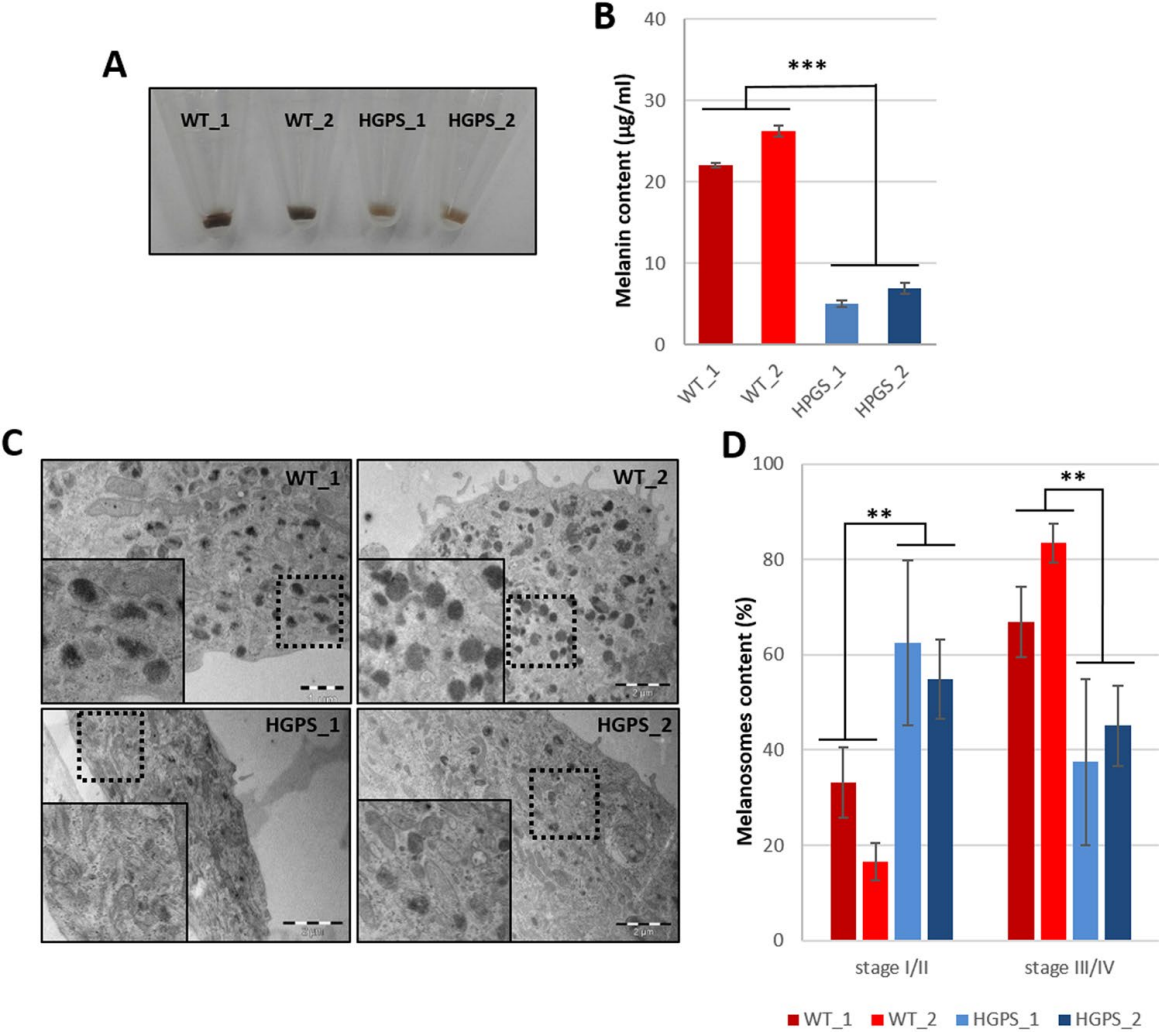
Characterization of pigmentation disorders and the melanosome maturation process in HGPS melanocytes. (**A**) Cellular pellet observation of WT (WT_1 and WT_2), HGPS (HGPS_1 and HGPS_2) Mel-iPSC. (**B**) Analysis of melanin content (optical density) in WT (WT_1 and WT_2), HGPS (HGPS_1 and HGPS_2) Mel-iPSC. Statistical analysis was performed with one-way analysis of variance (ANOVA), using Dunnet’s comparison test. Values of p < 0.05 were considered significant (*p < 0.05, **p < 0.01, ***p < 0.001). (**C**) Electron microscopy analysis of WT (WT_1 and WT_2), HGPS (HGPS_1 and HGPS_2) Mel-iPSC. An inset corresponding to a magnified area of the boxed region is shown on the left. (**D**) Quantification of melanosome stages (stage I/II and stage III/IV) in WT (WT_1 and WT_2), HGPS (HGPS_1 and HGPS_2) Mel-iPSC. Data are from three independent experiments. Statistical analysis was performed with one-way analysis of variance (ANOVA), using Dunnet’s comparison test. Values of p < 0.05 were considered significant (*p < 0.05, **p < 0.01, ***p < 0.001).

**Figure 5:**
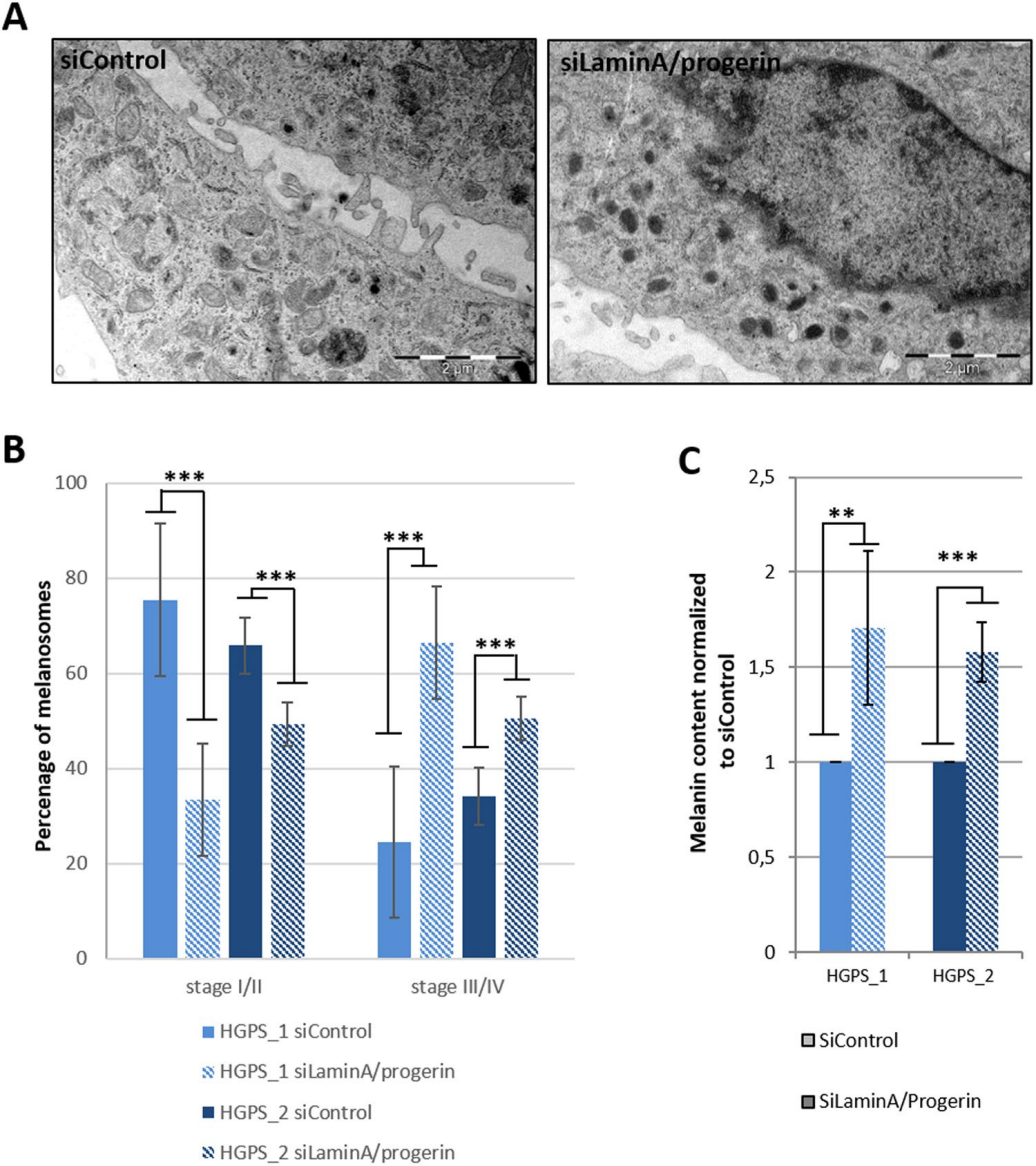
Progerin knockdown experiments revealed functional regulation of pigmentation in Mel-iPSC. (**A**) Electron microscopy analysis of HGPS Mel-iPSC transfected with siLaminA/Progerin and siControl. (**B**) Quantification of melanosome stages (stage I/II and stage III/IV) in HGPS (HGPS-1 and HGPS_2) melanocytes transfected with siLaminA/Progerin and siControl. Statistical analysis was performed with one-way analysis of variance (ANOVA), using Dunnet’s comparison test. Values of p < 0.05 were considered significant (*p < 0.05, **p < 0.01, ***p < 0.001). (**C**) Analysis of melanin content (optical density) in HGPS (HGPS_1 and HGPS_2), Mel-iPSC transfected with siLaminA/Progerin, normalized to siControl. Statistical analysis was performed with one-way analysis of variance (ANOVA), using Dunnet’s comparison test. Values of p < 0.05 were considered significant (*p < 0.05, **p < 0.01, ***p < 0.001).

Macroscopic observations of cell pellets containing HGPS and WT melanocytes revealed lower pigmentation in HGPS cells (Fig. 4A). Melanin quantification confirmed these observations, demonstrating that HGPS melanocytes produced five times less melanin than WT melanocytes, 21 µg/ml and 25 µg/ml vs 4 µg/ml and 6 µg/ml, respectively (Fig. 4B). The melanosome maturation process was analysed by electron microscopy in HGPS and WT melanocytes to investigate the cause of this deficient process of melanogenesis. Whereas all the stages of melanosome maturation were observed in all cell lines, whatever the genetic background (Fig. 4C), a higher proportion of non-mature melanosomes (type I and II) vs mature pigmented melanosomes (type III and IV) was observed in HGPS cells (Fig. 4C). Quantitative analysis of melanosome maturation stages revealed that the two HGPS melanocyte cell lines exhibit a significantly lower percentage of mature melanocytes (38% and 43%) than in WT cells (65% and 82%, respectively) (Fig. 4D). Conversely, a higher percentage of non-mature melanosomes was observed in HGPS cells (62% and 57%) than in WT cells (35% and 18%). Overall, these results reveal a deficient process of melanin production in HGPS melanocytes, suggesting a role of progerin in the regulation of melanosome maturation. In order to confirm this hypothesis, knockdown experiments were conducted using a siRNA that has previously been described to target progerin. While this sequence of siRNA was efficient up to 95% in skin fibroblasts, progerin was only partially decreased in melanocytes (40% in HGPS1 and 60% in HGPS2) (sup Fig C). Although incomplete, analysis of nuclear shape abnormalities confirmed a functional rescue of this phenotype (sup Fig D) following siRNA treatment. Analysis of the melanosome maturation process in HGPS melanocytes treated with the siRNA revealed a significant rescue of the hypopigmented phenotype (Fig. 5A), with a percentage of melanosome type III and IV close to that for WT melanocytes (Fig. 5B). In addition, measurements of melanin content confirmed this result, with a 60 to 70% increase in melanin in both HGPS melanocyte cell lines following 48 h of treatment with the siRNA (Fig. 5C) but no significant effect on the expression of MITF, TYRP1 and PMEL17 (sup Fig E and F).

## Discussion

We report here on a novel pathological mechanism associated with HGPS, showing that progerin regulates melanin synthesis and melanosome maturation in melanocytes derived from pluripotent stem cells. Our results shed new light on our understanding of the mechanisms underlying age-related skin dyspigmentation and describe a new potential readout that can be targeted in the search for potential treatment for HGPS, photo-aging and hypopigmentation disorders.

### Pathological modelling of HGPS using iPS cells

The pathological mechanism of HGPS and related premature aging has been extensively investigated over the past decade, mainly through the use of animal models or via the comparison of healthy vs HGPS primary cells (for a review, see^29^). These pioneer studies revealed that progerin accumulation disorganizes nuclear shape, leading to major consequences for cell proliferation, cell differentiation, the DNA repair process and chromatin organization. However, with a few exceptions, almost all of these observations were made on skin fibroblasts, thus limiting the exploration of their consequences for relevant tissue-specific functions. The use of iPS cells has hereby been the source of breakthrough discoveries for numerous genetic disorders, giving the research community access to an unlimited resource of cells based on the clinical features of the disease. In HGPS, the death of patients occurs at a mean age of 13 years due to cardiovascular problems caused by vascular dysfunction^4^. Analysis of vascular smooth muscle cells derived from iPS cells revealed premature senescence, increased apoptosis, an increase in calcification, a defective DNA repair process through defective DNAPK trafficking and suppression of PARP1 in HGPS VSMC that, jointly, could explain the loss of these cells in patient’s vessels^20,30,31^. Similarly, studies were performed on MSC derived from HGPS iPS cells to investigate the molecular mechanisms causing the loss of adipogenic and osteogenic tissues, describing a lipid storage defect in HGPS cells^23^ and an increase in the osteogenic differentiation process^13^, respectively. Here we describe that the presence of progerin leads to a decrease of melanin content in melanocytes derived from HGPS iPS cells suggesting an additional key role of progerin on the regulation of skin pigmentation. Although, there is no evidence that this effect is direct or indirect, our study revealed that melanosomes maturation process was partially rescued following progerin knockdown suggesting a functional relationship. Since 2011, the identifications of several other functional parameters in HGPS iPS cells derivatives has successively allowed to investigate the benefits of drug candidates on non-structural readouts, as demonstrated with rapamycin, lonafarnib, zoledronate or pravastatin^32^ or, more recentl with metformin^14^ and MG132^15^. Here, we describe a novel model associated with a new functional readout that can used for the further evaluation of new therapeutic strategies for patients with HGPS.

### HGPS, a model of physiological aging?

Aging is a complex process that results from several intrinsic mechanisms and exogenous factors operating simultaneously. Over the past decade, progerin has become a potential candidate for the regulation of this process, even though it cannot clearly be allocated to one single gene^33,34^. This hypothesis was proposed in 2006 because of experimental results showing the presence of progerin in cells^35,36^ and tissues of aged individuals, as described in skin^37^ and vessels^38^. These observations, in conjunction with the similarity of symptoms between HGPS and aged patients, highlight a potential link between ageing and progerin. The role of progerin in physiological aging has been intensively investigated, mainly through overexpression experiments in healthy cells, revealing similar dysregulation to that observed in cells from elderly individuals, with reduced proliferation and replicative senescence^39^, a decrease in cellular DNA repair capacity that leads to genetic instability^40,41^, a loss of telomeres^40^ and increased oxidative stress^42^. Taken together, these results and clinical observations suggest that progerin may at least contribute towards to physiological aging. In this context, the HGPS iPS cells and their derivatives offer a unique tool for consolidating the analogy between aging and HGPS and for modelling physiological aging.

### HGPS melanocytes to study pigmentation disorders associated with aging

Melanocytes are an essential cell type found in the skin and hair, providing pigmentation and photoprotection from ultraviolet (UV) radiation. The skin becomes mottled with hyper- and hypopigmentation areas as it ages, resulting in an irregular pattern of lighter and darker areas and spots called dyspigmentation and a reduced ability to tan^43^. Even if the molecular mechanism underlying skin aging is complex, several studies have reported that individuals appear paler with age due to a reduction in the number of melanocytes (8 to 20% per decade) and a reduced capacity to tan in response to UV radiation^44,45^. However, paradoxically, elderly patients usually present focal hyperpigmentation due to localized proliferation and aggregation of melanocytes^45^. Even though progerin expression in aged skin melanocytes remains unexplored to date, our results open up a new perspective for the understanding of the reduced pigmentation observed in the skin of HGPS patients and, by extension, in elderly individuals. To date, levels of progerin expression in melanocytes from elderly individuals remain unknown. However, if this hypothesis is supported, it would favour new dermatological indications for several compounds recently reported to decrease progerin expression, such as metformin, MG132 or retinoids.

## Material and Methods

### Fibroblast culture and reprogramming

HGPS Fibroblasts (13-8243) used in this study was isolated from a patient biopsy taken in the Assistance Publique Hôpitaux de Marseille during diagnostic procedures (referred as HGPS_1 in this study). HGPS Fibroblasts (AG001972) used in this study was provided by Coriell Cell Repository (Camden, USA) (referred as HGPS_2 in this study). Informed consents were obtained from the parents of the patient included in this study, complying with the ethical guidelines of the institutions involved and with the legislation requirements of the the french ministry of health (Declaration number DC-2008-429). The HGPS cell lines explored in this study have been prepared and stored according to the French regulation by the labeled Biological Resource Center of Tissues, DNA, Cells (CRB TAC), Department of Medical Genetics, la Timone Hospital, Marseille (Dr A De Sandre-Giovannoli and Mrs K. Bertaux). The fibroblast cell lines used belong to a biological sample collection declared to the French ministry of Health (Declaration number DC-2008-429) whose use for research purposes was authorized by the French ministry of Health (authorization numbers AC-2011–1312 and AC-2017-2986). Two control cell lines were used in this study. First cell line (DM4603) was provided by Coriell Cell Repository (Camden, USA) (referred as WT_1 in this study). Cells were obtained from the NINDS Human Genetics Resource Center DNA and Cell Line Repository. NINDS Repository sample numbers corresponding to the samples used are DM4603. The second control cell line (IMR-90) was obtained from ATCC (referred as WT_2 in this study). Cultures were maintained in Dulbecco’s modified Eagle’s medium + GlutaMAX II + 4500 mg/L D-Glucose (Gibco), supplemented with 20% fetal bovine serum (research grade, Sigma) and 1% sodium pyruvate 100 mM (Life technologies). Cell cultures were maintained at 37 °C, in 5% CO2 in a humidified atmosphere, with the media changed every 2 days. Fibroblasts from the four cell lines were reprogrammed into iPS cells using Yamanaka’s original method^46^ with OCT4, KLF4, SOX2, c-myc, and transferred using retroviral vectors. Molecular characterization of the four cell lines were already reported in a previous study^22^.

### Pluripotent stem cell culture and differentiation

WT and HGPS iPSCs were grown in colonies on mouse embryonic fibroblasts (MEF), inactivated with 10 mg/ml mitomycin C seeded at 30,000 cells/cm², and grown as previously described. For differentiation, embryonic bodies (EBs) are formed from hESC and iPSC clumps and grown on low attachment dishes in neural medium composed of 1/2 neurobasal and 1/2 HAM:F12 complemented with 2% of B-27 without VitA (Invitrogen) and 1% N-2 (Invitrogen). Induction of neural-crest differentiation was realized using CHIR-99021 (TOCRIS), LDN-193189 (Miltenyi) and SB 431542 (TOCRIS). Melanogenic commitment was realized with CHIR99021(TOCRIS), EDN3 (American peptide), SCF (peprotech), BMP-4 (peprotech) and ascorbic acid (Sigma-Aldrich). At day 7, EBs were plated on gelatine 0.1% and grown in MGM-4 medium supplemented with the same cytokines until around day 30.

### Melanocytes culture

Melanocytes derived from HGPS iPSCs and WT iPSCs were cultured in melanocytes specific medium, MGM-4 (Lonza). Cell cultures were maintained at 37 °C, in 5% CO2 in a humidified atmosphere, with the media changed every 2 days. Human adult epidermal melanocytes (HEM) were used as control.

### Quantitative PCR

Total RNA was isolated using an RNeasy Micro extraction kit (Qiagen, Courtaboeuf, France), according to the manufacturer’s protocol. An on-column DNase I digestion was performed to avoid genomic DNA amplification. RNA level and quality were checked using the Nanodrop technology. A total of 500 ng of RNA was used for reverse transcription using the Superscript III reverse transcription kit (Invitrogen). Q-PCR analysis was performed using a QuantStudio 12 K Flex real-time PCR system (Applied biosystem) and TaqMan gene expression Master Mix (Roche), respectively, following the manufacturers’ instructions. Quantification of gene expression was based on the DeltaCt Method and normalized to 18 S expression. PCR primers for 18 S, MITF, TYRP1, PMEL17 lamin A, lamin C and progerin were previously described^14,25,26^.

### Western immunoblotting

Whole-cell lysates of melanocytes were collected, proteins were extracted in NP40 cell lysis buffer (Invitrogen) with a protease and phosphatase inhibitor cocktail (ThermoScientific). Lysates were sonicated 4 times for 15 sec each, each with an interval of 15 sec in between, and then centrifuged at 10,000 g for 10 minutes at 4 °C. Protein concentration was measured using the Pierce BCA Protein Assay Kit (ThermoScientific) and the absorbance at 562 nm was evaluated using a Clariostar (BMG Labtech). A total of 20 µg of protein was loaded and run on a 7% tris-acetate gel (Criterion™ XT) using XT tricine running buffer (Biorad). Gels were then transferred onto polyvinylidene fluoride (PVDF) membranes (Biorad) using a Trans-Blot Turbo Transfer System (Biorad). Blots were blocked in Rockland blocking buffer (Millipore) diluted 1:2 in TBS 1× for 1 hour at room temperature. Membranes were incubated with primary antibodies diluted in blocking buffer with 0.1% Tween20 (VWR) overnight at 4 °C. The primary antibodies used here are a mouse anti-progerin 1:200 (SantaCruz, 13A4D4, sc-81611) and a mouse anti-actin 1:5,000 (Millipore, MAB1501R). Washing was carried out for 45 minutes at room temperature with TBS + 0.1% Tween20 and the membranes were incubated with an IR-Dye 800CW conjugated with a secondary donkey anti-mouse antibody at 1:10,000 in blocking buffer with 0.1% Tween20 and 0.01% SDS (Ambion). The detector was an Odyssey Infrared Imaging System (LI-COR Biosciences).

### Immunocytochemistry

Cells were fixed in 4% paraformaldehyde (15 minutes, room temperature) before permeabilization in PBS supplemented with 0.1% triton X-100 (Sigma) (5 minutes, room temperature). They were then blocked during 30 minutes at room temperature using PBS with 1% BSA (Sigma-Aldrich, St. Louis, USA). The primary antibodies used are: Mouse anti-MITF (1:100, M362129-2, Dako), Rabbit anti-lamins A/C (1:200, ab26300, Abcam), Mouse anti-NKI (1:100, ab34165, Abcam), Mouse anti-TYRP1 (1 :500, LSC39939)were incubated for one hour at room temperature in blocking buffer. Cells were stained with the species-specific fluorophore-conjugated secondary antibody (Invitrogen) (one hour, room temperature) and nuclei were visualized with Hoechst 33342 (Invitrogen).

### SiRNAs transfection

Melanocytes were transfected with SiControl or SiLamninA/Progerin using Oligofectamine (Invitrogen). Cells were transfected a second time after 48 h and analysed after 96 h from the first shot of transfection. siRNA Lamin-Progerin : 5′-GCAUCUAUCUCAUCUAUCU-3′/ 5′-AGAUAGAUGAGAUAGAUGC-3′.

### Melanin content analysis

100 000 cells were lysed in 1 M NaOH (Sigma-Aldrich®), warmed at 65 °C during 2 H and then centrifuged. Absorbance of supernatants was measured at 405 nm using 96-well microplates. A standard synthetic melanin curve (0 to 50 μg/ml) was performed in triplicate for each experiment (Sigma-Aldrich®).

### Electron Microscopy

For ultrathin cryosectioning, melanocytes were fixed with 2% PFA or with a mixture of 2% PFA and 0.2% glutaraldehyde in 0.1 M phosphate buffer, pH 7.4. Cells were processed for ultracryomicrotomy (70 nm of thickness) and stained using the method described for ultrathin cryosections^47^. All samples were analysed using a FEI CM120 electron microscope (FEI Company), and digital acquisitions were made with a numeric camera (Keen View; Soft Imaging System, SIS, Germany).

### Image Analysis and quantification

Quantification of melanosomes was determined using the iTEM software (Soft Imaging System, SIS, Germany).

### Statistical analysis

Statistical analysis was performed with one-way analysis of variance (ANOVA), using Dunnet’s comparison test. Values of p < 0.05 were considered significant (*p < 0.05, **p < 0.01, ***p < 0.001).

## Electronic supplementary material

Supplementary Figure
