# Supplementary material for: Pathological modelling of pigmentation disorders associated with Hutchinson-Gilford Progeria Syndrome (HGPS) revealed an impaired melanogenesis pathway in iPS-derived melanocytes
Source: Sci Rep. 2018 Jun 14;8:9112. doi: 10.1038/s41598-018-27165-y (PMC6002548; doi:10.1038/s41598-018-27165-y)
Supplement: Supplementary file 1 — Supplementary Figure [file 41598_2018_27165_MOESM1_ESM.pdf]

Supplementary information

Pathological modelling of pigmentation disorders associated with Hutchinson-Gilford Progeria Syndrome (HGPS) revealed an impaired melanogenesis pathway in iPS-derived melanocytes

Authors: Alessandra Lo Cicero[1], Manoubia Saidani[1], Allouche Jennifer[1], Anne Laure Egesipe[1], Lucile Hoch[1], Celine Bruge[1], Sabine Sigaudy[2,3], Annachiara De Sandre-Giovannoli[2,3], Nicolas Levy[2,3], Christine Baldeschi[1], Xavier Nissan[1*]

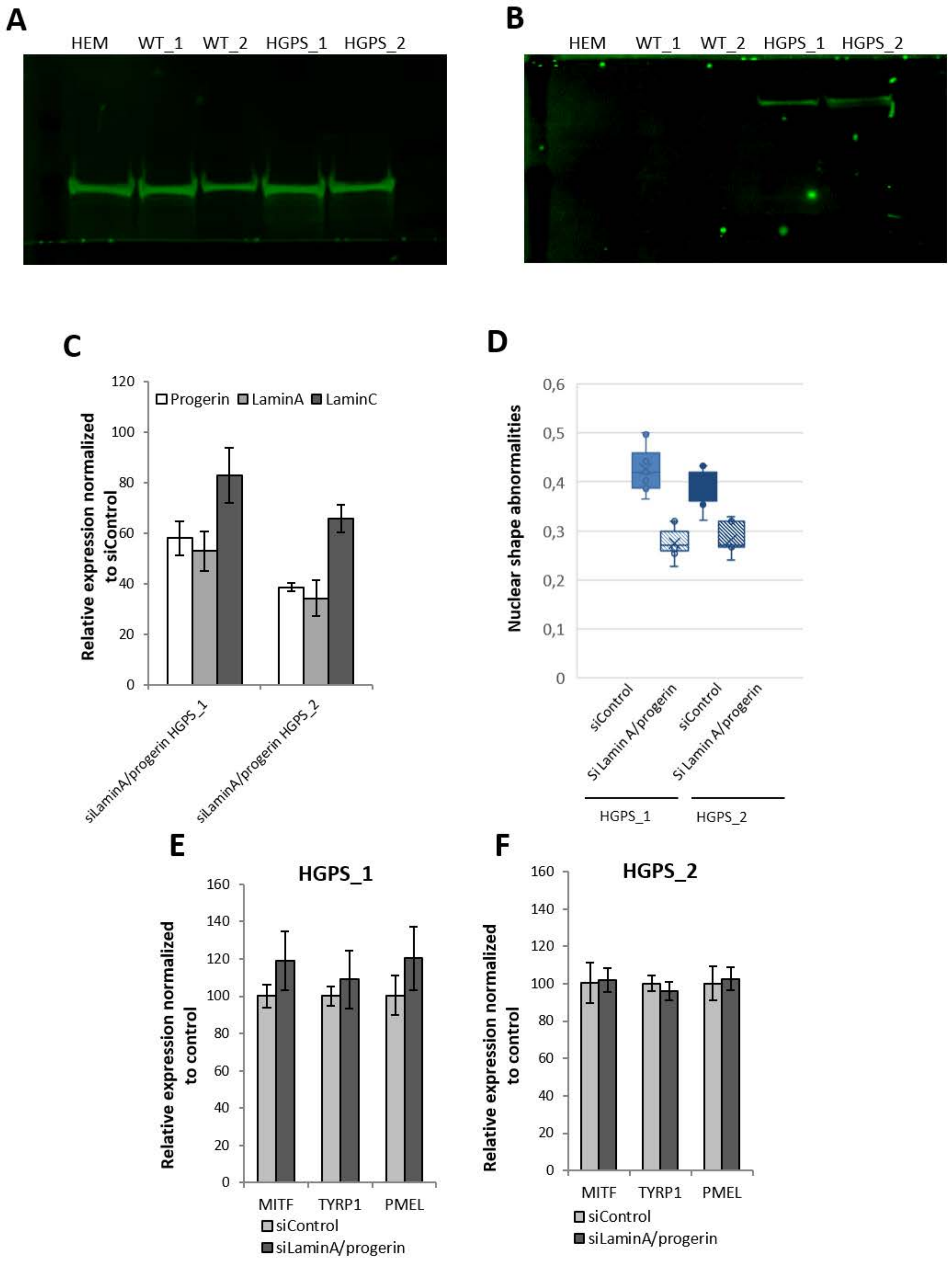
A
HEM
WT_1
WT_2
HGPS_1
HGPS_2
B
HEM
WT_1
WT_2
HGPS_1
HGPS_2
C
Relative expression normalized to siControl
Progerin
LaminA
LaminC
120
100
80
60
40
20
0
siLaminA/progerin HGPS_1
siLaminA/progerin HGPS_2
D
Nuclear shape abnormalities
0,6
0,5
0,4
0,3
0,2
0,1
0
siControl
Si Lamin A/progerin
siControl
Si Lamin A/progerin
HGPS_1
HGPS_2
E
HGPS_1
Relative expression normalized to control
160
140
120
100
80
60
40
20
0
MITF
TYRP1
PMEL
siControl
siLaminA/progerin
F
HGPS_2
Relative expression normalized to control
160
140
120
100
80
60
40
20
0
MITF
TYRP1
PMEL
siControl
siLaminA/progerin

SUPP FIGURE LEGEND

A) Uncropped Actin blots corresponding to figure 2C
B) Uncropped progerin blots corresponding to figure 2C
C) Measurement of progerin, lamin A and lamin C expression in HGPS (HGPS_1 and HGPS_2) Mel-iPSC transfected with siLaminA/Progerin. Results were normalized to siControl.
D) Analysis of nuclear shape abnormalities in HGPS (HGPS_1 and HGPS_2) Mel-iPSC transfected with siLaminA/Progerin. Results were normalized to siControl.
E) Measurement of MITF, TYRP1 and PMEL17 in HGPS_1 Mel-iPSC transfected with siLaminA/Progerin. Results were normalized to siControl.
F) Measurement of MITF, TYRP1 and PMEL17 in HGPS_2 Mel-iPSC transfected with siLaminA/Progerin. Results were normalized to siControl.
